# Genomic Sequence of Mycobacteriophage OKCentral2016

**DOI:** 10.1128/genomeA.01208-17

**Published:** 2018-02-22

**Authors:** Christopher James Patton, Hari Kotturi

**Affiliations:** a Department of Biology, University of Central Oklahoma, Edmond, Oklahoma, USA

## Abstract

OKCentral2016 is the first mycobacteriophage sequenced from Oklahoma soil using the bacterial host *Mycobacterium smegmatis* strain mc^2^155. OKCentral2016 has a double-stranded DNA genome composed of 50,072 bp, with 84 predicted coding genes and 1 tRNA sequence. This mycobacteriophage has sequence similarities to members of the A10 subcluster.

## GENOME ANNOUNCEMENT

OKCentral2016 was isolated from enriched topsoil at the University of Central Oklahoma (35.653371 N, 97.474118 W) using the bacterial host *Mycobacterium smegmatis* mc^2^155. The plaques produced by this phage were clear and had a diameter of approximately 4 mm after 24 h of incubation. High-titer phage lysate was obtained by using polyethylene glycol 8000 and the NaCl salt precipitation method (1, 2). Electron microscopy revealed a *Siphoviridae* morphotype, consisting of an icosahedral head that was approximately 60 nm in diameter and a tail that was approximately 120 nm in length. The genome was sequenced using Illumina (Solexa) next-generation sequencing with the MiSeq platform. The genome was assembled using Newbler and Consed software. OKCentral2016 has a genome that was 50,072 bases, with a GC content of 65.1%.

The genome of OKCentral2016 was annotated using DNA Master (http://cobamide2.bio.pitt.edu/). Protein-coding regions of the genome were detected using Glimmer (3) and GeneMark (4). Starterator (https://seaphages.org/software/) was used to evaluate gene start codons. Gene function was determined using Phamerator (5) and BLASTn/BLASTp on PhagesDB.org. ARAGORN (6) and tRNAscan-SE (7) were used to analyze tRNA sequences within the genome. OKCentral2016 was shown to possess 84 protein-coding genes and 1 tRNA (tRNA^Trp^) sequence that was 76 bases in length. There were two programmed frame shifts that were observed. We also detected programmed frameshifts in the tail assembly chaperone and in the primase gene.

OKCentral2016 is the first mycobacteriophage sequenced from Oklahoma soil. The OKCentral2016 genome contains 3′ cohesive ends, which are 10 bases in length. The 3′ overhang sequence was shown to be 5′-CGGCCGGTAA-3′. The GC contents of the host bacterium, *M. smegmatis* mc^2^155, and OKCentral2016 were similar, at 67.4% and 65.1%, respectively (8). OKCentral2016 was shown to align most with two other A10 subcluster phages, Chupacabra and Goose, by using a BLASTn search on PhagesDB.org with an expected value of 0.0 and a minimum identity value of 99%. OKCentral2016 shared a strong syntenic region coding for lysin A, lysin B, terminase, portal, capsid maturation protease, scaffolding, and major capsid subunit proteins with Goose and Twister, other A10 subcluster mycobacteriophages.

### Accession number(s).

The genome sequence of OKCentral2016 is available from GenBank under the accession no. MF773750.
